# Nitric Oxide Inhibits Al-Induced Programmed Cell Death in Root Tips of Peanut (*Arachis hypogaea* L.) by Affecting Physiological Properties of Antioxidants Systems and Cell Wall

**DOI:** 10.3389/fphys.2017.01037

**Published:** 2017-12-21

**Authors:** Chun-Liu Pan, Shao-Chang Yao, Wei-Jiao Xiong, Shu-Zhen Luo, Ya-Lun Wang, Ai-Qin Wang, Dong Xiao, Jie Zhan, Long-Fei He

**Affiliations:** ^1^College of Agronomy, Guangxi University, Nanning, China; ^2^College of Life Science and Technology, Guangxi University, Nanning, China; ^3^Guangxi Botanical Garden of Medicinal Plants, Nanning, China; ^4^Key Laboratory of Crop Cultivation and Tillage, Guangxi Colleges and Universities, Nanning, China

**Keywords:** nitric oxide, Al stress, programmed cell death, antioxidant defense, cell wall

## Abstract

It has been reported that nitric oxide (NO) is a negative regulator of aluminum (Al)-induced programmed cell death (PCD) in peanut root tips. However, the inhibiting mechanism of NO on Al-induced PCD is unclear. In order to investigate the mechanism by which NO inhibits Al-induced PCD, the effects of co-treatment Al with the exogenous NO donor or the NO-specific scavenger on peanut root tips, the physiological properties of antioxidants systems and cell wall (CW) in root tip cells of NO inhibiting Al-induced PCD were studied with two peanut cultivars. The results showed that Al exposure induced endogenous NO accumulation, and endogenous NO burst increased antioxidant enzyme activity in response to Al stress. The addition of NO donor sodium nitroprusside (SNP) relieved Al-induced root elongation inhibition, cell death and Al adsorption in CW, as well as oxidative damage and ROS accumulation. Furthermore, co-treatment with the exogenous NO donor decreased MDA content, LOX activity and pectin methylesterase (PME) activity, increased xyloglucan endotransglucosylase (XET) activity and relative expression of the xyloglucan endotransglucosylase/hydrolase (*XTH-32*) gene. Taken together, exogenous NO alleviated Al-induced PCD by inhibiting Al adsorption in CW, enhancing antioxidant defense and reducing peroxidation of membrane lipids, alleviating the inhibition of Al on root elongation by maintaining the extensibility of CW, decreasing PME activity, and increasing XET activity and relative *XTH-32* expression of CW.

## Introduction

Aluminum (Al) toxicity is the main reason for large-scale decline of arable land. In acidic soils with pH lower than 4.5, Al becomes the soluble Al^3+^ ion, released into soil solution and resulting in plant Al stress, which subsequently affects a plant's ability to take up water and nutrients; as a consequence, Al toxicity is among the most important factors limiting crop growth and production (Matsumoto et al., 2015). It has been estimated that approximately 40% of the world's arable soil is acidic. Previous reports have revealed the mechanism of Al response and tolerance in model plants like rice (Ma et al., 2002), wheat (Kabir et al., 2011), rye (Gallego and Benito, 1997), and *Arabidopsis* (Ezaki et al., 2001). However, there is an urgent need to understand the mechanism of Al tolerance in other plants, to help us to solve the problems of food shortage and inadequate biofuel production from acid soils (Sun et al., 2015).

Programmed cell death (PCD) is a gene-controlled process of cell suicide, which may play an important role in Al tolerance. PCD is involved in plant growth, development and response to various environmental stresses. It has been suggested that the mechanism of PCD induction usually includes an increase in the local levels of reactive oxygen species (ROS) under abiotic stress (Petrov et al., 2015; Zabka et al., 2017). Al-induced PCD is one of the major causes of Al toxicity in plants (Panda et al., 2008; Li and Xing, 2010; He et al., 2015). Our previous studies showed that there was a negative relationship between PCD and Al tolerance, and that the exogenous nitric oxide (NO) donor sodium nitroprusside (SNP) inhibited Al-induced PCD occurrence (Zhan et al., 2013; Huang et al., 2014a,b; He et al., 2017).

The cell wall (CW) is the first site where contact is made with Al, and it plays a pivotal role in the perception and manifestation of Al toxicity (Chang et al., 1999; Yang et al., 2007; Horst et al., 2010). The relative abilities of different CW components (pectin, hemicellulose 1 [HC1] and hemicellulose 2 [HC2]) to adsorb Al has been studied in recent years (Yang et al., 2011a,b). For instance, pectin content was positively correlated with Al-induced cell death in maize suspension cells (Schmohl and Horst, 2000). The pectin content of the root apex in an Al-sensitive cultivar was higher than in an Al-tolerant cultivar in rice (Yang et al., 2007). Al treatment resulted in a significant increase in pectin methylesterase (PME) activity after 6 h compared to the control (Sun et al., 2016). These results indicate that the binding of Al to pectin is an important step in Al tolerance. However, several studies reported that hemicellulose (HC) metabolism was more sensitive to Al stress than pectin. For example, exposure of Scout 66 (an Al-sensitive wheat cultivar) to low Al treatment for 6 h resulted in the accumulation only of HC but not pectin (Tabuchi and Matsumoto, 2001). Similarly, Yang et al. (2007) reported that the most significant change of a CW component under Al stress in rice was HC. Approximately 75% of the CW-bound Al after exposure to 50 μM Al for 24 h in *Arabidopsis* accumulated in the HC1 component (Yang et al., 2011a). Al might bind to multiple sites of the CW, and the binding sites may be different in different plants.

The extractable xyloglucan endotransglucosylase (XET) activity and xyloglucan endotransglucosylase/hydrolase (*XTH*) gene expression as cell growth promoters have often been correlated with cell expansion (Vissenberg et al., 2000, 2003, 2005; Osato et al., 2006; Van Sandt et al., 2007; Zhu et al., 2012; Lurlaro et al., 2016). The *Arabidopsis* genome contains 33 different *XTH* genes which are response to environmental stress, such as cold/heat shock (Xu et al., 2010), flooding (Saab and Sachs, 1995), drought stresses (Lurlaro et al., 2016) and Al stress (Zhu et al., 2012; Shi et al., 2015).

As an important signal transduction molecule, NO is involved in a variety of physiological and molecular processes in plants (Gow and Ischiropoulos, 2001). The diverse biological functions of NO in plants vary, depending on cell types, cellular localization, concentration and species. NO has been shown to play an important role in the stress response of plants to heavy metals, including cadmium (Ma et al., 2010), arsenic (Leterrier et al., 2012), and copper (Hu et al., 2015). It has been reported that NO could reduce Al toxicity by alleviating Al-induced inhibition of root growth, preventing Al-induced oxidative stress (Wang and Yang, 2005; Tian et al., 2007; Wang et al., 2010). Treatment of the seedlings with the NO donor SNP with Al mitigated the mitochondrial respiratory dysfunction induced by Al stress in wheat root tips (He et al., 2006). SNP (200 μmol·L^−1^) inhibited Al-induced PCD, while 50 μmol·L^−1^ 2-4-carboxylphenyl-4, 4, 5, 5-teramethylimidazoline-1-oxy-3-oxide (cPTIO, a NO-specific scavenger) promoted Al-induced PCD (He et al., 2017). Considering the role of the CW in Al toxicity, NO is an important endogenous signal in interfering with CW properties and affecting the capacity of CW to bind to Al (Xiong et al., 2009; Zhang et al., 2011; Sun et al., 2016). However, the corresponding physiological and molecular mechanisms which NO alleviates Al-induced PCD remain elusive. The modification of properties of the CW and antioxidants systems is the main issue to understanding the function of NO in Al-induced PCD. In order to investigate the mechanism by which NO inhibits Al-induced PCD, the effects of co-treatment Al with the exogenous NO donor or the NO-specific scavenger on peanut root tip growth were studied, and the properties of antioxidants systems and CW in cultivars differing with respect to Al tolerance were compared under the conditions of Al-induced PCD.

## Materials and methods

### Plant material and growth condition

Seeds of peanut cultivars 99-1507 (Al-tolerant) and ZH2 (Al-sensitive) were prepared as described by Zhan et al. (2008) with slight modifications. After incubating in moistened perlite for 3–4 days in the dark at 26 ± 1°C, seedlings with roots 2–3 cm long were transferred to Hoagland nutrient solution, which was renewed every 2 days with fresh nutrient solution. After the emergence of the fourth leaf, the seedlings were pretreated for 24 h in 0.1 mM CaCl_2_ (pH 4.2) solution. In the time-course treatment, the seedlings were exposed to 100 μM AlCl_3_ (containing 0.1 mM CaCl_2_, pH 4.2) for 0, 4, 8, 12, 24h, respectively. For elucidating the mechanism of NO on alleviating Al toxicity, seedlings were exposed to the following treatments for 12 h as follows: 100 μM AlCl_3_ (containing 0.1 mM CaCl_2_, pH 4.2); 100 μM AlCl_3_ and 200 μM SNP (containing 0.1 mM CaCl_2_, pH 4.2); 100 μM AlCl_3_ and 50 μM cPTIO (containing 0.1 mM CaCl_2_, pH 4.2); 200 μM SNP (containing 0.1 mM CaCl_2_, pH 4.2); 50 μM cPTIO (containing 0.1 mM CaCl_2_, pH 4.2); 0.1 mM CaCl_2_ (pH 4.2) (control). All seedlings were grown in an illuminated incubator at 26 ± 1°C, with a light intensity of 30–50 μmol·m^−2^·s^−1^. Freshly- treated root tips (1.0 ± 0.1 cm) were collected for the following experiments.

### Assay of relative root growth and cell death

The root elongation from different treatments was determined by measuring the main root length and expressed as relative root elongation (RRE). The RRE was calculated follow the formula: (L_12h_-L_0h_) × 100%/L_0h_· L_12h_ represented the root length for 12 h treatment, L_0h_ represented the initial root length before treatment. Cell death of root tips was determined by the method described by Huang et al. (2014a). In brief, root tips (1.0 ± 0.1 cm) were stained with 0.25% (w/v) aqueous solution of Evans blue for 15 min, and then rinsed with distilled water for 30 min. After the stained root tips were immersed in 4 ml *N, N*-dimethylformamide for 1 h, the solution was collected (containing the dye which had leached from the dead cells) and the absorbance was measured at 600 nm.

### 4′,6-diamidino-2-phenylindole (DAPI) staining

Fresh treated root tips (approximately 1 cm) were fixed in 70% osmotically balanced formaldehyde-acetate-alcohol fixative solution buffer for more than 12 h. After root tips were rinsed in ddH_2_O three times, dehydrated using an increasing ethanol series (70, 80, 85, 90, 95, and 100%, 1 h for each step), and permeabilized in xylene. The tips were processed and embedded in paraffin wax. The root tips were cut into longitudinal section (10 μm thick) with fully motorized rotary microtome (RM2250, Leica) and adhered to glass microscope slides. Serial tissue sections were deparaffinized in xylene, and rehydrated through a decreasing alcohol series. They were dyed by 1 mg·L^−1^ DAPI in ddH_2_O for 30 s, and observed using a fluorescence microscope (BX53, Olympus, Japan) at 385–400 nm excitation.

### Hematoxylin staining for *in vivo* Al detection

Freshly- treated root tips (1.0 ± 0.1 cm) were rinsed in distilled water for 15 min to wash away the Al residue on the surface. After staining with 0.1% (w/v) hematoxylin (containing 0.01% (w/v) KIO_3_) for 20 min, the root tips were soaked in distilled water for 10 min, and then observed with a stereo microscope (SZX7, Olympus, Tokyo, Japan).

### Determination of Al content in root tips and in CW

For determination of total Al content of the root tips, excised root tips (1.0 ± 0.1 cm root segments) were placed in a 1.5 ml eppendorf tube containing 1.0 ml 2 M HCl, as described by Osawa and Matsumoto (2001). The tubes were incubated at 25°C for 24 h, with occasional shaking to release Al from the root tips; the suspending solution was collected and used to determine total root tip Al concentration. CW material was prepared by the method described by Zhong and Lauchli (1993), with minor modifications. Root tips were ground with a mortar and pestle in liquid nitrogen and then homogenized with 7 ml 75% alcohol for 20 min in an ice bath. The sample was then centrifuged at 13,000 × g for 15 min and the supernatant was discarded. The pellets were washed with precooled (4°C) acetone, methanol: chloroform (1:1), and methanol. After drying in an oven at 60°C for 12 h, the pellet was suspended in 1 ml 2 M HCl at room temperature for 24 h with occasional shaking. The Al concentration in the extracts of root tips and CW were determined by inductively-coupled Plasma Optical Emission Spectrometry (ICP-OES; Varian700, Agilent, Santa Clara, California, USA). To clarify the difference of Al enrichment, the percentage of Al content in CWs to total Al in root tips was determined by calculating the ratio of Al content in CWs to the total Al in 1.0 g fresh root tips.

### Al adsorption and desorption kinetics assay

The Al adsorption and desorption kinetics assay was carried out according to the method described by Zheng et al. (2004), with some modifications. A total of 10 mg CW material was placed into a 2 ml column equipped with a filter at the bottom, the adsorption solution (consisted of 100 μM AlCl_3_ in 0.1 mM CaCl_2_ at pH 4.2) was run through the column using a peristaltic pump set at a speed of 4 mL per 20 min. The adsorption solution was collected at 20 min intervals. The Al adsorbed was desorbed by 0.5 mM CaCl_2_ at pH 4.2 at the same speed as adsorption. The accumulative Al adsorbed or desorbed was measured spectrometrically according to Kerven et al. (1989).

### Determination of NO content

NO in peanut root tips was detected by the specific NO fluorescent probe DAF-FM diacetate (Beyotime, Haimen, China) as described previously (Corpas et al., 2009; Yang et al., 2013). After incubation at 25°C for 1 h in the dark with 10 μM DAF-FM diacetate, root tips were washed twice with distilled water. A fluorescence image was taken with a confocal laser scanning microscope (Leica TCS SL, Leica Microsystems, Heidelberg, Germany) at an excitation of 488 nm and emission of 515 nm.

### Estimation and staining of H_2_O_2_ and superoxide radical (${\text{O}}_{2}^{\cdot -}$)

*In situ* detection of ${\text{O}}_{2}^{\cdot -}$ was performed as described by Dunand et al. (2007) with slight modifications. Root tips were stained for 15 min in a freshly prepared solution of 1 mM nitroblue tetrazolium (NBT) in 20 mM phosphate buffer (pH 6.5). *In situ* staining of H_2_O_2_ in root tips was carried out according to the method of Yang et al. (2014) with some modifications. Root tips were incubated in a freshly-prepared solution of 3, 3′-diaminobenzidine (DAB, 1 mg·ml^−1^) for 4 h. After staining for either ROS, root tips were washed three times with water and observed with a stereomicroscope (Leica DM4000B).

The concentrations of H_2_O_2_ and ${\text{O}}_{2}^{\cdot -}$ in root tips were measured as described by Sergiev et al. (1997) and Liu et al. (2013), respectively.

### Estimation of lipid peroxidation

The level of lipid peroxidation was assayed by measuring the malondialdehyde (MDA) content, according to the method of Cheng et al. (2015), with some modifications. Approximately 0.2 g root tips were homogenized in 10 ml trichloroacetic acid (TCA) and centrifuged at 13,000 × g for 10 min. An aliquot (2 ml) of the supernatant was mixed with 2 ml 0.6% (w/v) thiobarbituric acid in 10% TCA and heated in a boiling water bath for 15 min. The mixture was quickly cooled in an ice bath and then centrifuged at 13,000 × g for 10 min. The absorbance of the supernatant was determined at 450, 532, and 600 nm. The MDA concentration (C) was estimated using the formula as follows: C (nmol mL^−1^) = 6.45 × (A532–A600) − 0.56×A450.

### Assay of antioxidant enzyme activities

Root tips (0.2 g) were homogenized with a mortar and pestle, using 50 mM sodium phosphate buffer (pH7.8), containing 1 mM EDTA, 2% (w/v) PVP, 1 mM PMSF, 1 mM DTT and 0.05% (v/v) Triton X-100. The homogenate was centrifuged at 13,000 × g at 4°C for 15 min and the supernatant was used for determination of enzyme activities. Protein concentration of enzymes extracts was measured by Bradford Protein Assay Kit (Beyotime, Haimen, China), using bovine serum albumin (BSA) as standard.

Lipoxygenase (LOX) activity was determined according to the method of Surrey (1964), with some modifications. The enzyme activity were determined by mixing 50 μl root tip extract with 2.75 ml potassium phosphate buffer (pH 6.5) and 0.2 ml 7.5 mM linoleic acid containing 0.25% (v/v) Tween 20. LOX activity was proportional to the absorbance at 234 nm.

Superoxide dismutase (SOD) activity was measured according to Qureshi et al. (2013) by measuring the inhibition of photochemical reduction of NBT at 560 nm. Catalase (CAT) activity was determined following the method of Hossain et al. (2010) by determining the decomposition of H_2_O_2_ and monitoring the decrease of absorbance at 240 nm for 3 min. Peroxidase (POD) activity was determined by measuring the rate of guaiacol oxidation and monitoring the absorbance at 470 nm, according to Choudhary (2011). Ascorbate peroxidase (APX) activity was determined according to the method of Asada (1984), measuring the rate of ascorbate oxidation at 290 nm.

### Determination of PME and XET activity

PME was extracted according to the method of Rodoni et al. (2010). Root tips (0.2 g) were homogenized with 2 ml 1 M NaCl containing 1% (w/v) polyvinylpolypyrrolidone, and then centrifuged at 13,000 × g for 20 min at 4°C. The supernatant was collected and used for assaying the enzyme activity. The activity was assayed in a mixture containing 1 ml 0.5% (w/v) pectin (pH7.5), 0.4 ml 0.01% (w/v) bromothymol blue (pH7.5), 1.55 ml distilled water (pH7.5) and 50 μl root tip extract. Activity was determined following the method of Hagerman and Austin (1986), measuring the absorbance at 620 nm.

XET activity was detected *in situ* according to the method of Vissenberg et al. (2000). Root tips were incubated for 1 h in 6.5 μM xyloglucan oligosaccharides (XGO-SRs) dissolved in 2 mM MES buffer. Root tips were washed for 10 min in ethanol: formic acid: water (15:1:4, v/v/v) to remove unreacted XGO-SRs, and then incubated in 5% formic acid to remove apoplastic, non-wall-bound XGO-SRs. These samples were observed by confocal laser scanning microscope (Leica TCS SL, Leica Microsystems) with excitation at 552 nm and emission at 580 nm.

### *XTH-32* gene expression assay

The expression of *XTH-32* was analyzed by real-time fluorescent quantitative PCR (qRT-PCR). *XTH-32* expression data were presented relative to the expression level of the *UBQ10R* gene (NCBI accession number: EG030441). RNA was extracted from root tips (0–10 mm) using the Trizol method (Catalog No. 15596-018; Invitrogen, Carlsbad, CA, USA) following the manufacturer's instructions. The cDNA was synthesized from 1 μg total RNA using RevertAid TM first Strand cDNA Synthesis Kit (Catalog No. 1621; Fermentas, Waltham, MA, USA). The primers for *XTH-32* were 5′-ATTGAGTTTCTTGGGACTACGTTTG-3′ (forward) and 5′- ATTTGACCATCTCCACTTCCTCT-3′ (reverse). The primers for *UBQ10R* were 5′-CGCACACTCGCTGACTACAAC-3′ (forward) and 5′-CACGGAGACGGAGGACAAGG-3′ (reverse).

## Statistical analysis

The data were subjected to data processing with Excel 2007 (Microsoft Inc., Redmond, WA, USA) and analyzed using SPSS v.13.0 (SPSS Inc., Chicago, IL, USA). The summary statistics presented in the figures are means ± SD of three replicates for each sample; Bars with different lowercase letters are significantly different at *P* < 0.05, as determined by ANOVA. The figures were drawn using the software Graphpad Prism 5 (GraphPad software, San Diego, CA, USA).

## Results

### Effects of exogenous NO on Al-induced root elongation growth and cell death in root tips of peanut

**Figure 1:**
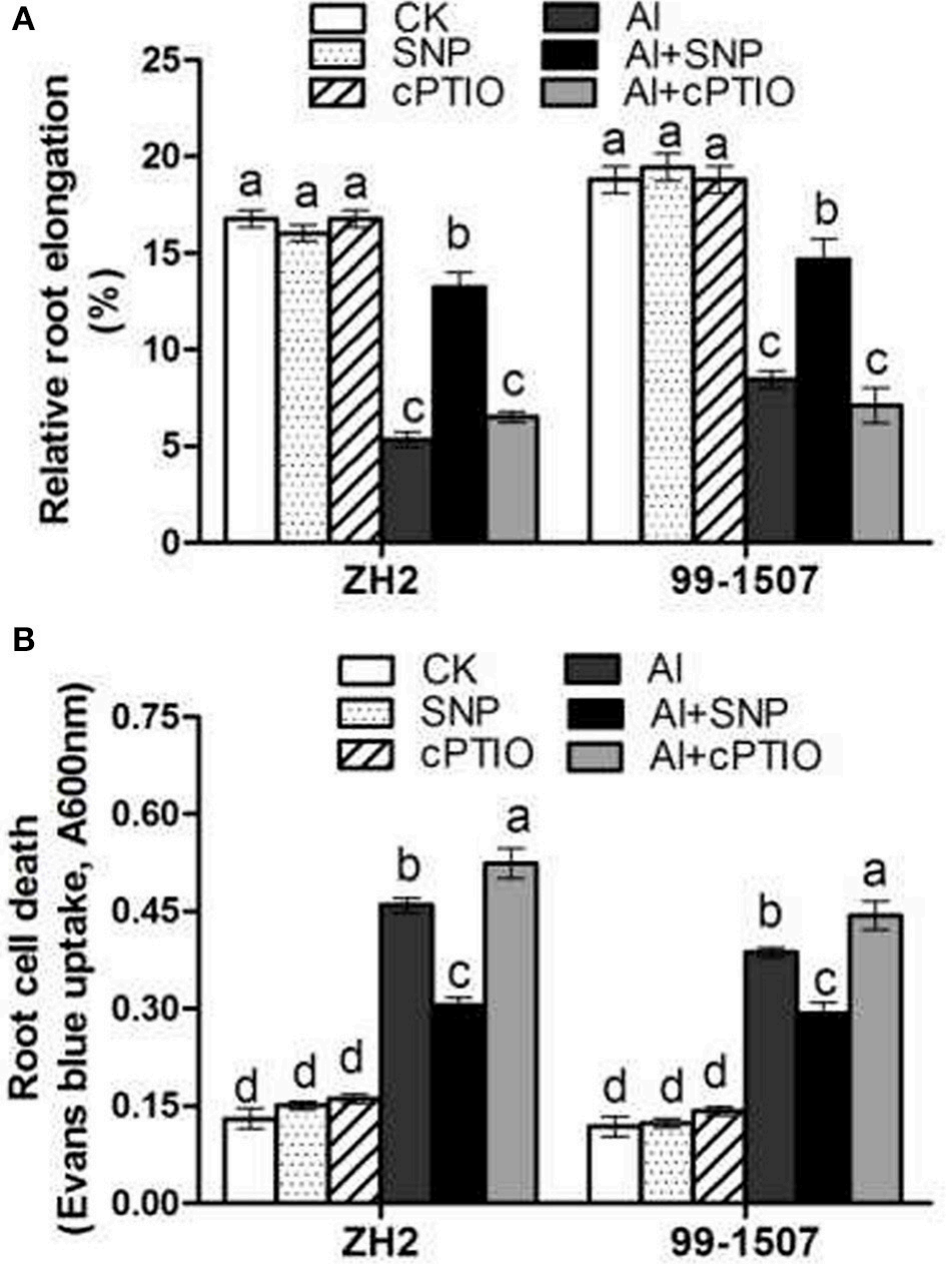
Effects of exogenous NO on Al-induced root elongation growth and cell death in root tips of ZH2 and 99–1507 exposed to 100 μM Al treatment for 12 h. **(A)** Effects of Al, SNP, and cPTIO on root elongation (CK = control). **(B)** Effects of Al, SNP, and cPTIO on cell death. Values represent means ± *SD* (*n* = 3). Different letters indicate significant differences at *P* < 0.05.

Al significantly inhibited root length of peanut. The inhibition by Al on root elongation of Al-sensitive cultivar ZH2 was greater than that of 99-1507. Compared to the corresponding no-Al control, the root length of ZH2 and 99-1507 under Al stress was 31.9 and 45.0%, respectively (Figure 1A). SNP and cPTIO alone had no effect on root elongation compared to the control. However, co-treatment Al with SNP effectively alleviated the inhibition of root growth induced by Al stress in two peanut genotypes, with the relative root lengths in the Al+SNP treatments being 79.0% (ZH2) and 78.1% (99-1507), compared to the Al treatment alone. But co-treatment Al with cPTIO, a specific NO scavenger, did not significantly change the inhibition of root elongation, compared with Al treatment alone (Figure 1A).

The integrity of the cell membrane during Al stress was evaluated using a non-permeable dye, Evans blue. Compared to the control, SNP and cPTIO alone had no effect on root tip cell death. 100 μM AlCl_3_ increased significantly root tip cell death in both cultivars. However, cell death in root tips of ZH2 was 18.8% higher than that of 99-1507 under Al tress. Al-induced cell death was significantly reduced by the addition of SNP, while the addition of cPTIO greatly increased cell death by aggravating the damage to cell membranes (Figure 1B).

### DAPI staining

**Figure 2:**
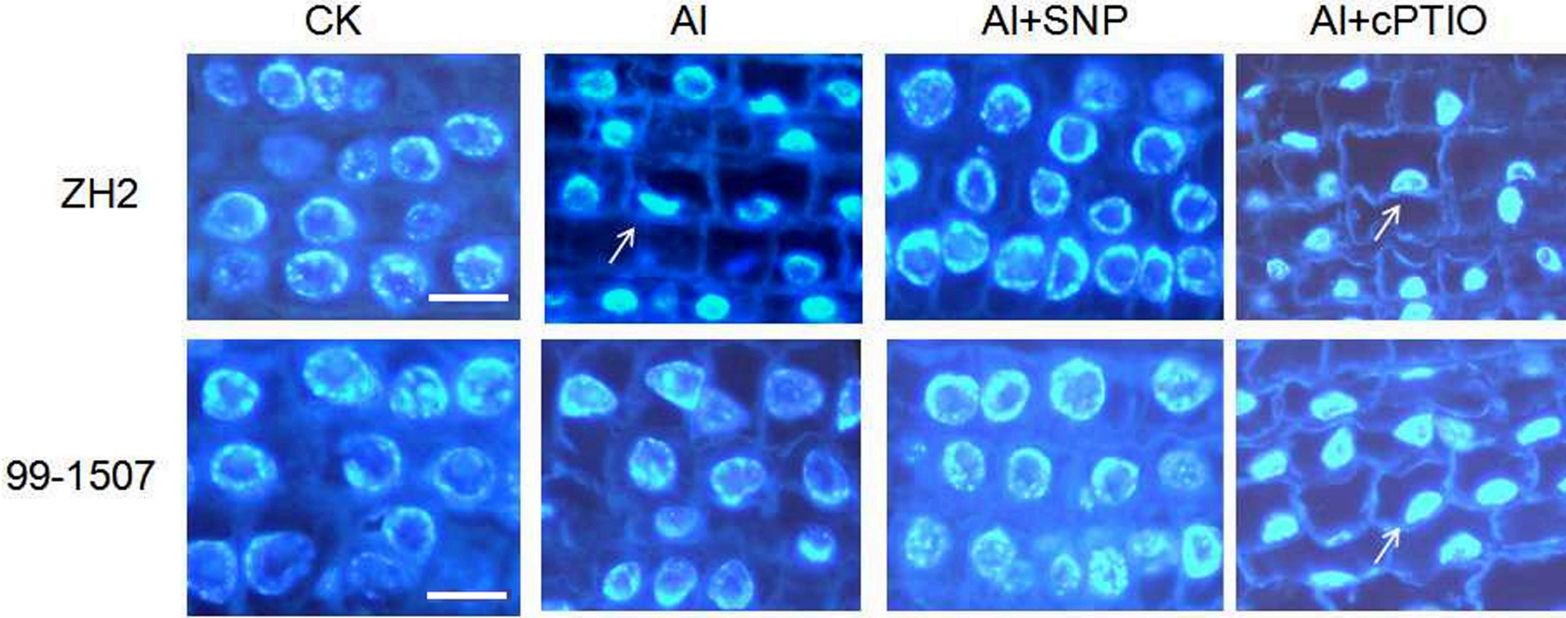
The DAPI staining in root tip cells of ZH2 and 99–1507 under different treatments. Bar = 25 μm.

To further investigate nuclear changes, the cells were stained with DAPI and examined under a fluorescent microscope after different treatments. The nucleus of normal cell is usually spherical and locates in the middle of the cell without Al stress. Nuclei exposed to Al were flattened, lobed, invaginated or irregular in shape. As shown in Figure 2, NO could prevent PCD caused by Al toxicity. Compared with Al treatment alone, the nuclear remained round after the addition of exogenous NO donor SNP. However, the integrity of cell structure was lost and nuclear condensation presented small crescent-shaped after the addition of NO specific scavenger cPTIO under Al treatment.

Compared with Al treatment alone, NO reduced nuclear deformation, it was verified that exogenous NO inhibited Al-induced PCD in root tip cells of peanut. The effect of cPTIO was in contrast to NO, which confirmed that NO played a negative role in the regulation of PCD induced by Al.

### Effects of exogenous NO on Al accumulation in root tips and CW

**Figure 3:**
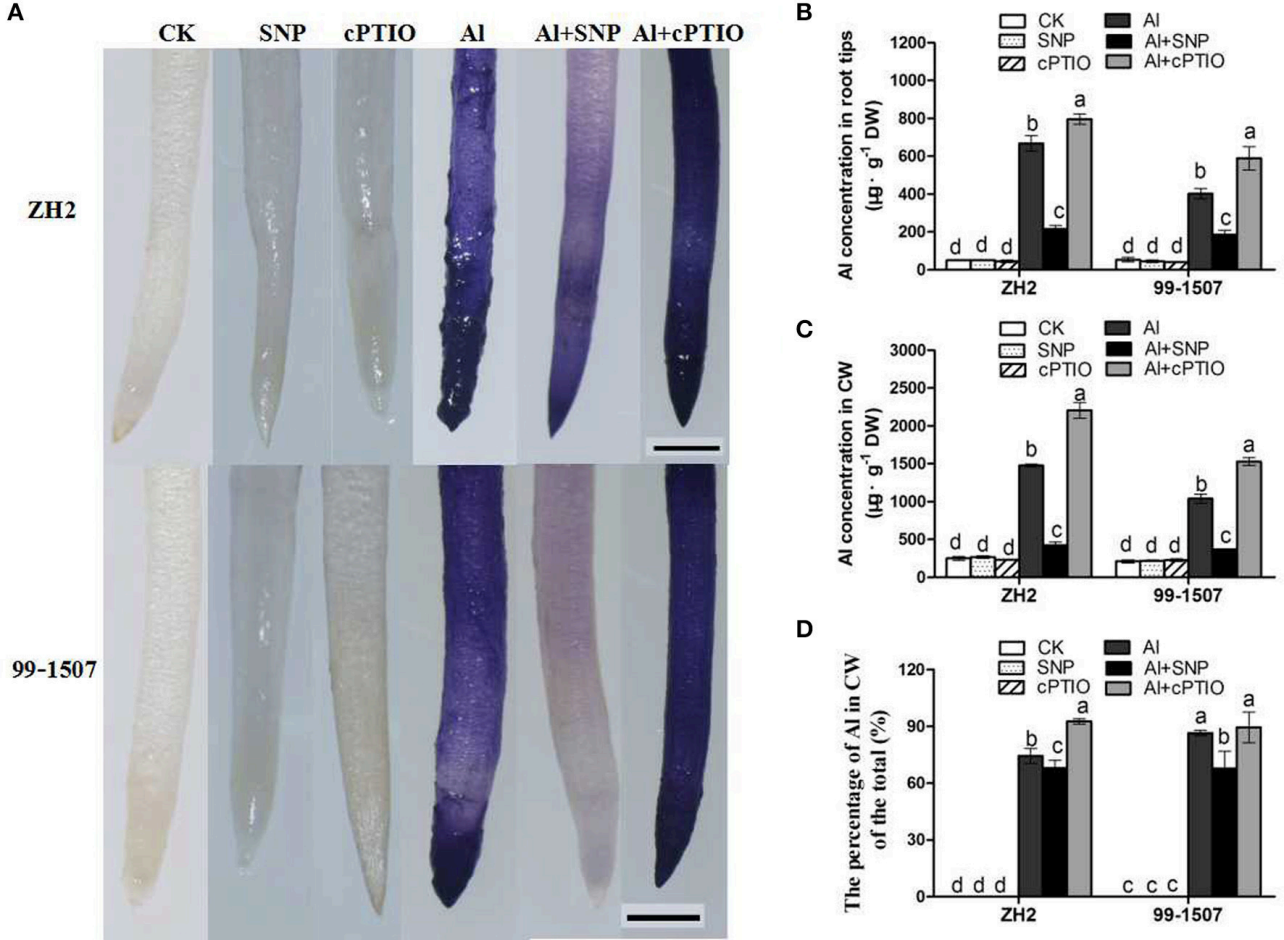
Effects of exogenous NO on Al accumulation in root tips and CW of ZH2 and 99–1507 with 100 μM Al treatment for 12 h. **(A)** Effects of Al, SNP, and cPTIO on hematoxylin staining to detect in situ Al accumulation. **(B)** Effects of Al, SNP, and cPTIO on Al concentration in root tips. **(C)** Effects of Al, SNP, and cPTIO on Al concentration in root tip CW. **(D)** Effects of Al, SNP, and cPTIO on the percentage of Al content in CWs to total Al in root tips. Values represent means ± *SD* (*n* = 3). Different letters indicate significant differences at *P* < 0.05. Scale bars = 200 μm.

The degree of hematoxylin staining (to visualize Al accumulation) in the root tips of ZH2, exposure to 100 μM Al treatment for 12 h was deeper than that in 99-1507, indicating that Al accumulation was more greater in the Al-sensitive ZH2 (Figure 3A). Compared with Al treatment alone, co-treatment Al with SNP significantly decreased cell staining in root tips of both peanut cultivars, but the effect was more obvious in 99-1507 than in ZH2. Co-treatment Al with cPTIO increased the accumulation of Al in the root tips (Figure 3A).

Al accumulation in peanut root tips and in the root tip CW was detected by ICP-OES. As shown in Figures 3B,C, a significant increase in Al accumulation in root tips and in CWs was observed after Al treatment. Compared with Al treatment alone, co-treatment Al with SNP significantly decreased Al accumulation in root tips and in CWs. On the other hand, co-treatment Al with cPITO significantly increased Al concentration in the root tip CW of ZH2, but not in 99-1507. In the meanwile, there was more Al accumulation in CWs in 99-1507 than in ZH2, and co-treatment Al with SNP decreased the percentage of Al content in CWs to total Al in root tips (Figure 3D). Compared to the control, SNP and cPTIO alone had no effect on the accumulation of Al in the root tips, CW and the percentage of Al content in CWs to total Al in root tips (Figures 3A–D).

### Al adsorption and desorption kinetics of CW

**Figure 4:**
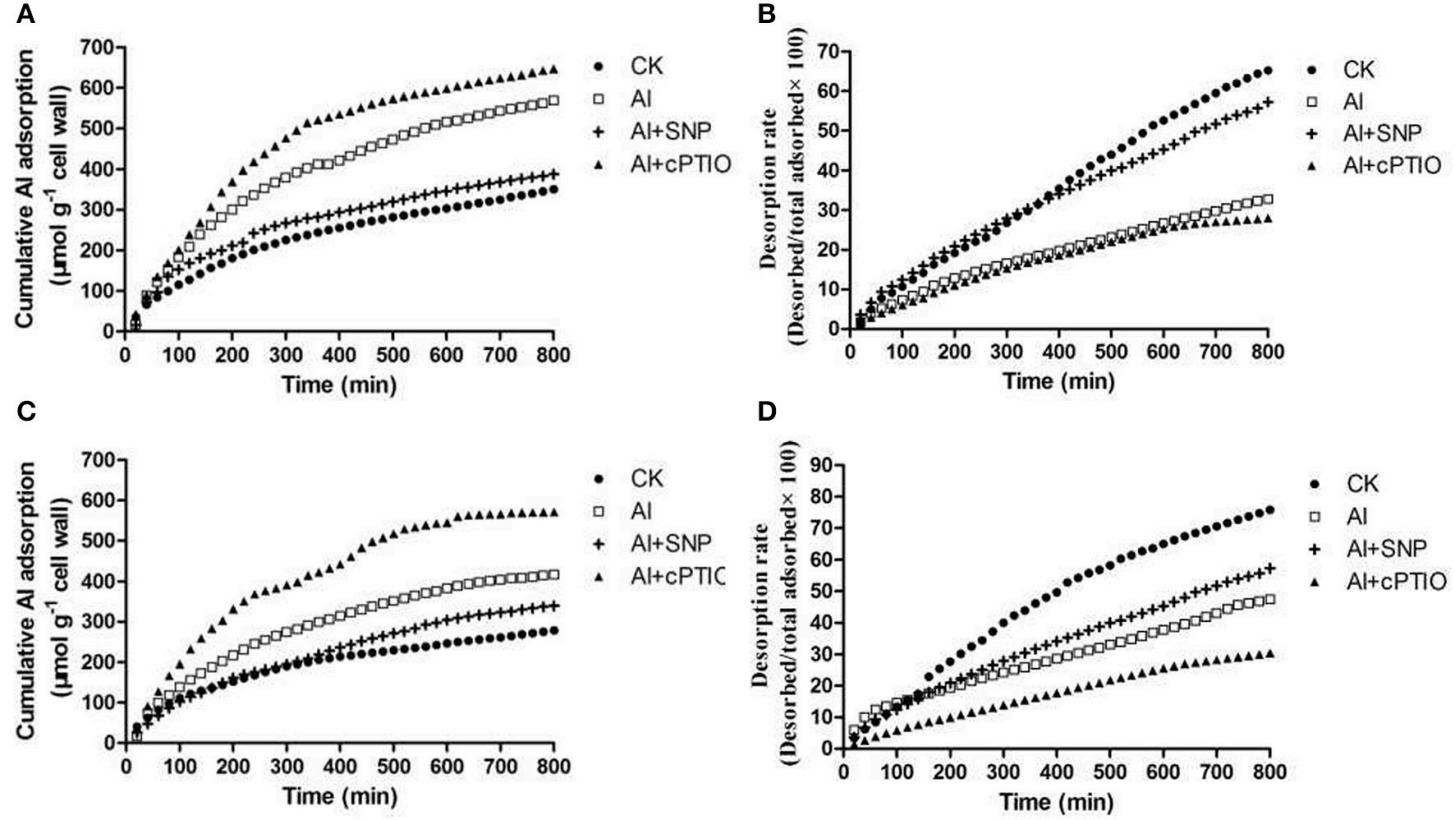
Adsorption and desorption kinetic curves of Al in root tip CWs of ZH2 and 99–1507. **(A)** Effects of Al, SNP, and cPTIO on Al adsorption in root tip CW of ZH2. **(B)** Effects of Al, SNP, and cPTIO on Al desorption in root tip CW of ZH2. **(C)** Effects of Al, SNP, and cPTIO on Al adsorption in root tip CW of 99–1507. **(D)** Effects of Al, SNP, and cPTIO on Al desorption in root tip CW of 99–1507. At least two independent replicates were conducted and one set of results was presented.

Al adsorption and desorption experiments in CWs of two varieties without Al treatment demonstrated that CW of ZH2 had high ability to accumulate Al, it was 25.6% greater in ZH2 than in 99-1507 (Figures 4A,C). Only 65.1% of the total Al absorbed in CW of ZH2 was desorbed within 800 min, whereas the desorption rate of 99-1507 was up to 75.8%, indicating that the ability of CW binding to Al is related to Al tolerance in peanut (Figures 4B,D). After 12 h of Al treatment, the total Al absorbed in the root tip CW of ZH2 and 99-1507 increased by 67.1 and 49.6% respectively, compared to without Al treatment (Figures 4A,C). Compared to Al treatment alone, the addition of SNP decreased 33.8 and 18.5% of the total Al adsorption in CW of ZH2 and 99-1507, respectively, whereas the addition of cPTIO increased the Al adsorption ability in the CWs of ZH2 and 99-1507 (Figures 4A,C). After 12 h of Al treatment, the total Al desorbed in the root tip CW of ZH2 and 99-1507 decreased by 50.8 and 37.3% respectively, compared to without Al treatment (Figures 4B,D). Compared to Al treatment alone, the addition of SNP increased 78.1 and 21.3% of the total Al desorption in CW of ZH2 and 99-1507, respectively, whereas the addition of cPTIO to Al decreased the Al desorption ability in the CWs of ZH2 and 99-1507 (Figures 4B,D).

### The production of endogenous NO in peanut root tips under Al stress

**Figure 5:**
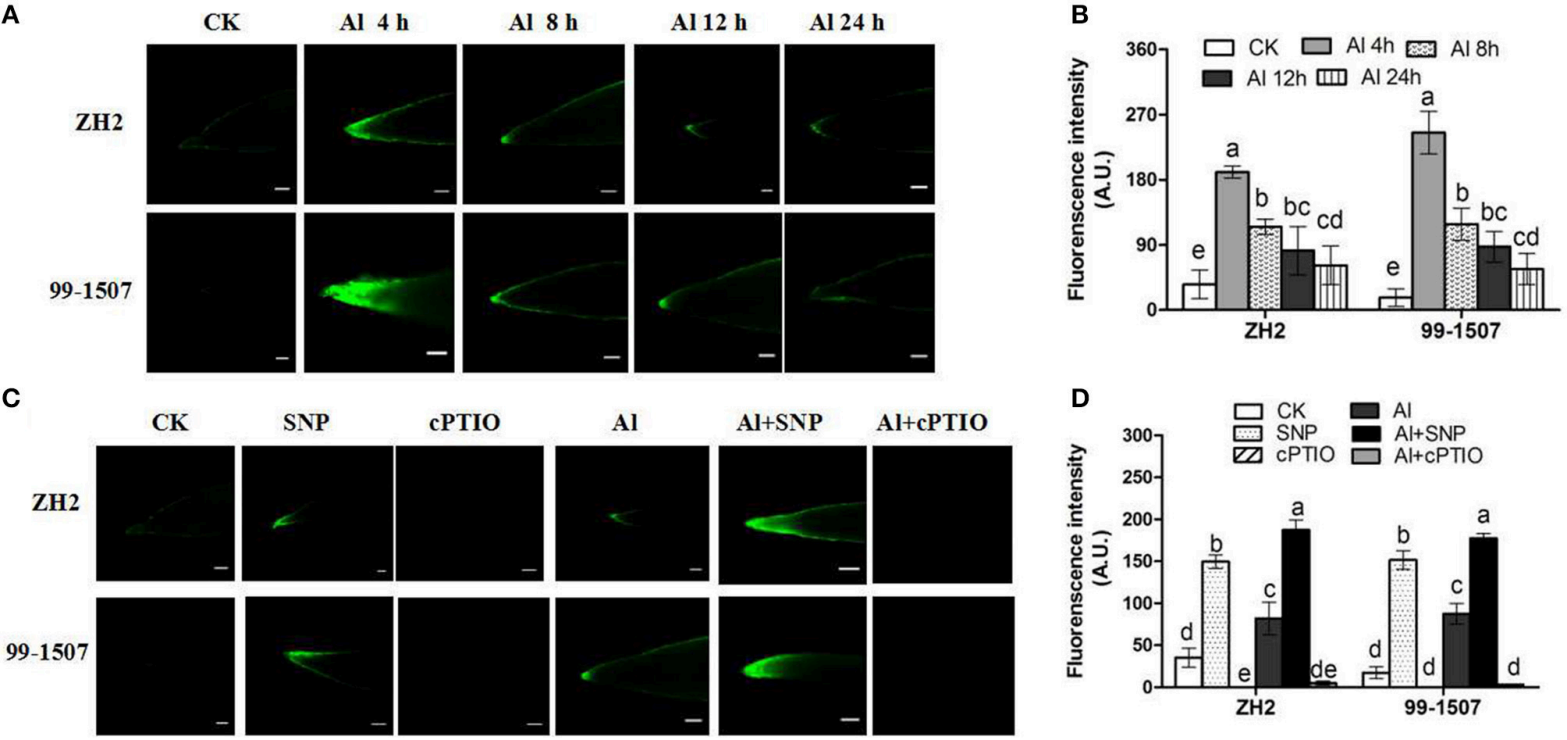
Fluorescence detection of peanut root tips by DAF-FM diacetate staining and confocal microscopy. Scale bars = 100 μm. Fluorescence intensity was expressed as arbitrary units (A.U.) using Image J software (Rawak Software Inc., Germany). **(A)** Fluorescence detection of ZH2 and 99–1507 under different Al treatments times. **(B)** Fluorescence intensity of ZH2 and 99–1507 under different Al treatments. **(C)** Fluorescence detection of ZH2 and 99–1507 by addition of SNP and cPTIO. **(D)** Fluorescence intensity of ZH2 and 99–1507 by addition of SNP and cPTIO. Values represent means ± *SD* (*n* = 3). Different letters indicate significant differences at *P* < 0.05.

The production of endogenous NO in peanut root tips was examined using the permeable NO-sensitive dye fluorophore DAF-FM diacetate. The addition of NO donor SNP under no-Al tress showed a strong fluorescence, indicating that SNP could be catalyzed and released NO in peanut root tips (Figure 5A). Compared to the control, a stronger fluorescence was observed in the root tips of ZH2 and 99-1507 following Al treatment. The results showed that Al exposure induced endogenous NO accumulation, and an early burst of NO at 4 h in the root tips of ZH2 (Al-sensitive) and 99-1507 (Al-tolerant), with fluorescence intensity in ZH2 and 99-1507 being 5.4- and 14.1-fold higher than that of the corresponding no-Al treatment (Figures 5A,B). However, with the increase of Al treatment time, the fluorescence in root tips of two cultivars decreased. Co-treatment Al with SNP significantly increased NO level in root tips of the two cultivars, NO production by ZH2 and 99-1507 being 2.3- and 2.0-fold higher, than that of the Al treatment alone, respectively (Figures 5C,D). Co-treatment Al with 50 μM cPTIO effectively inhibited the production of endogenous NO (Figures 5C,D).

### Effects of Al and NO on ROS accumulation and enzyme activities

**Figure 6:**
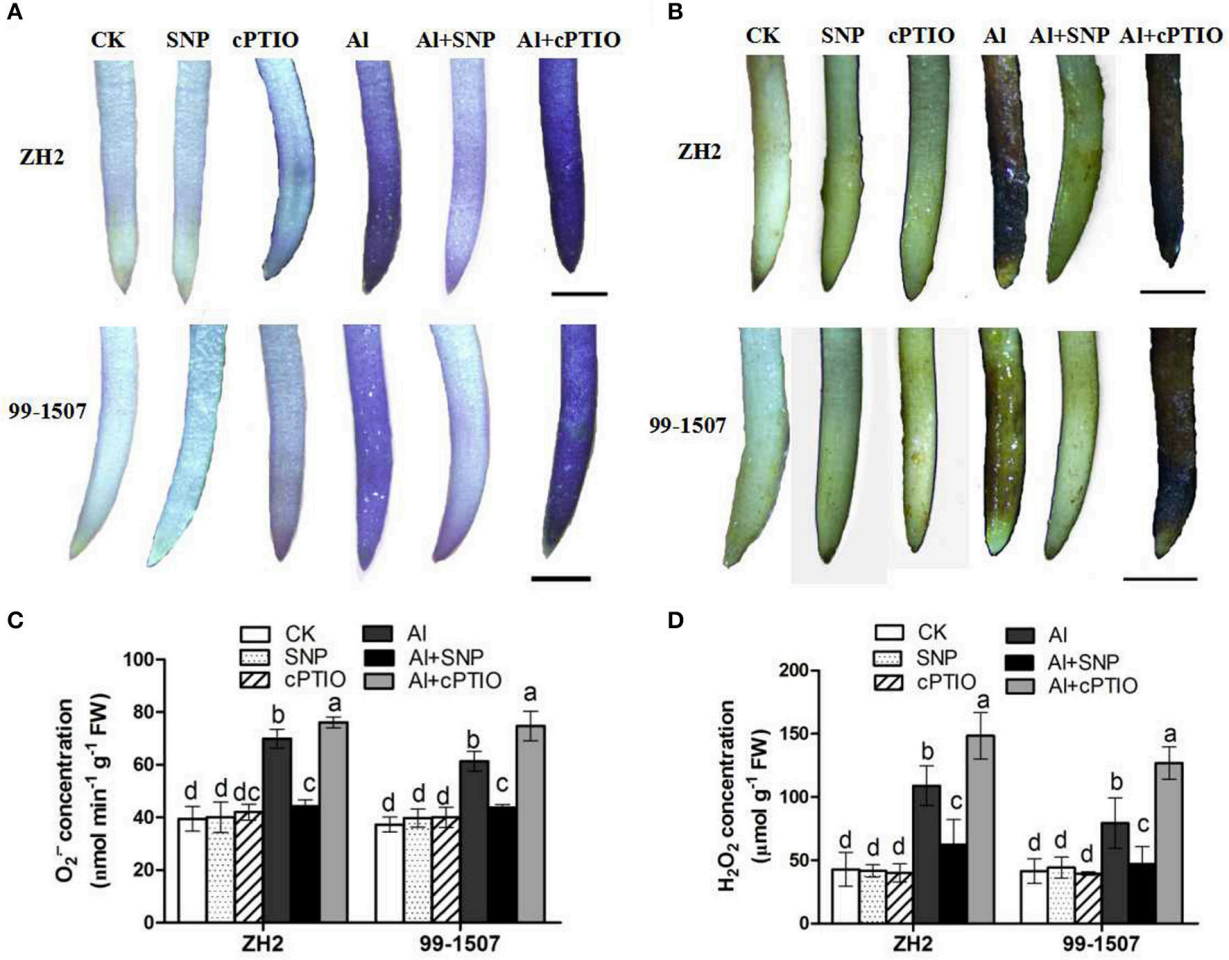
Changes in the accumulation of ${\text{O}}_{2}^{\cdot -}$ and H_2_O_2_ in root tips of peanut under different Al treatments. **(A)** *In situ* detection of ${\text{O}}_{2}^{\cdot -}$ in root tips of ZH2 and 99–1507 by DAB staining. **(B)** *In situ* detection of H_2_O_2_ in root tips of ZH2 and 99–1507 by NBT staining. Scale bars = 200 μm. **(C)** The concentration of ${\text{O}}_{2}^{\cdot -}$ in root tips of ZH2 and 99–1507. **(D)**The concentration of H_2_O_2_ in root tips of ZH2 and 99–1507. Values represent means ± *SD* (*n* = 3). Different letters indicate significant differences at *P* < 0.05.

The generation of the ROS, ${\text{O}}_{2}^{\cdot -}$ and H_2_O_2_ *in situ* in peanut root tips under different treatments was detected histochemically, using NBT and DAB, respectively. Compared with the control, root tips were stained deeply with both NBT and DAB under Al stress (Figures 6A,B). The root tips of ZH2 and 99-1507 stained weakly following the Al+SNP treatment, but stained extensively following the Al+cPTIO treatment. It was found that the generation of both ${\text{O}}_{2}^{\cdot -}$ and H_2_O_2_ in root tips of the Al-sensitive ZH2 was significantly higher than that of the Al-tolerant 99-1507 under Al stress, indicating higher levels of Al-induced oxidative stress in root tips of ZH2 (Figures 6C,D). Adding SNP significantly decreased the ROS concentration (${\text{O}}_{2}^{\cdot -}$ and H_2_O_2_) in both cultivars, but co-treatment Al with cPTIO increased significantly the ROS concentration. SNP alone and cPTIO alone had no significant effect on the ROS accumulation of two cultivars, compared with the control (Figures 6A–D).

### Effects of NO on MDA concentration and LOX activity

**Figure 7:**
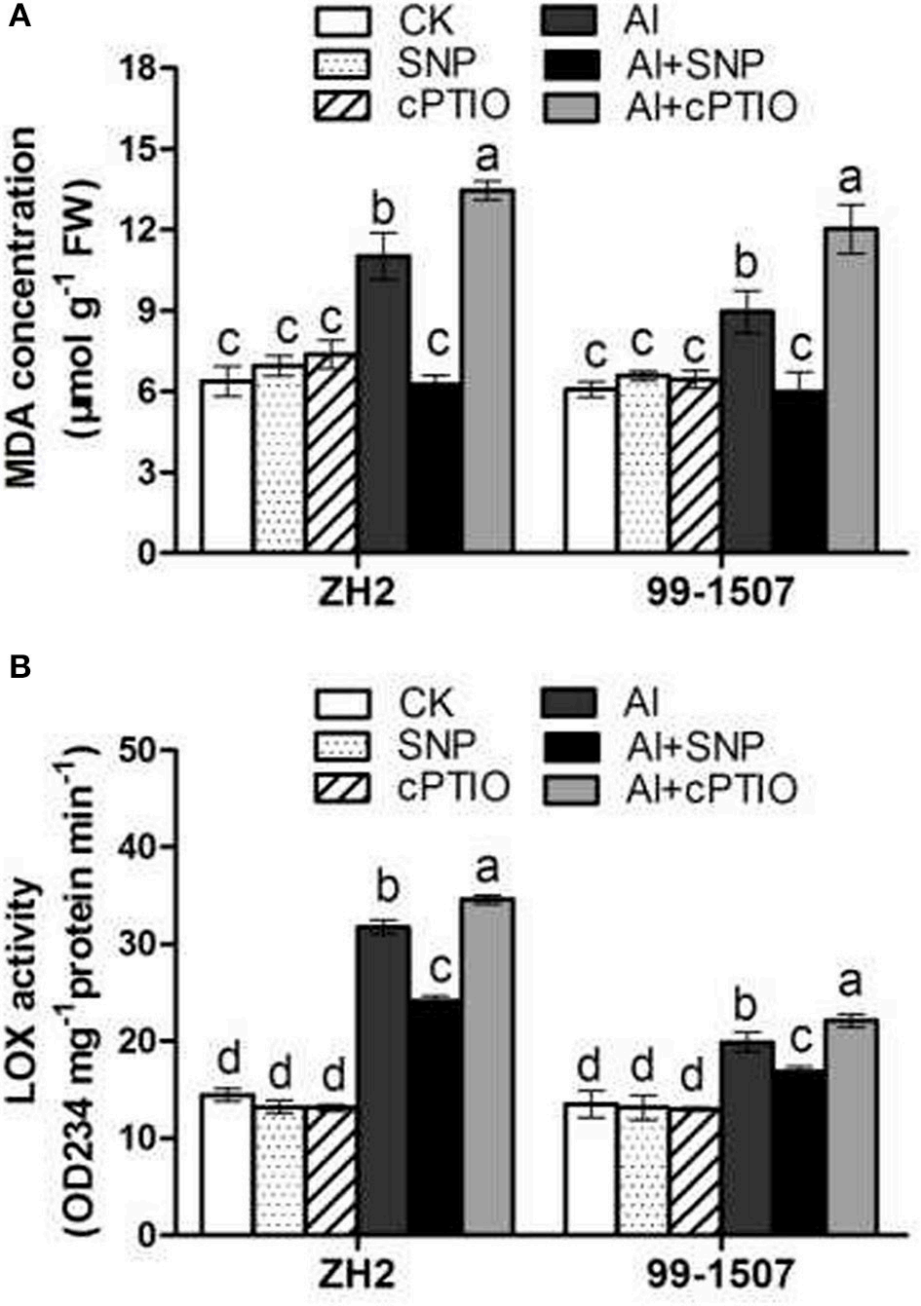
Effects of exogenous NO on oxidative damage parameters in root tips of ZH2 and 99–1507 with 100 μM Al treatment for 12 h. **(A)** MDA concentration; **(B)** LOX activity. Values represent means ± *SD* (*n* = 3). Different letters indicate significant differences at *P* < 0.05.

The concentration of MDA is an indicator of lipid peroxidation and oxidative damage to membranes, while LOX activity is involved in catalyzing the formation of H_2_O_2_ derivatives and activating the lipid peroxidation of membranes. The increase in MDA concentration and LOX activity in the Al-stress treatment showed that Al caused severe oxidative damage to plasma membranes in both cultivars. Figure 7A showed that Al treatment caused a significant increase (84.8% higher than the control) in MDA concentration in ZH2 whereas it was only increased by 69.3% in the Al-tolerant 99-1507. Co-treatment Al with SNP clearly inhibited the increase of MDA content induced by Al stress in both cultivars, but the Al+cPTIO treatment increased significantly MDA levels compared to Al alone. LOX activity in ZH2 and 99-1507 increased by 119.8% and 47.5%, respectively, following exposure to 12 h Al stress. Significant differences were observed between LOX activities in the two cultivars at all the treatments, with ZH2 having the higher activities, except for the control. SNP decreased LOX activity, but cPTIO increased LOX activity in both cultivars to above the activities observed in the Al stress treatment (Figure 7B). Compared with the control, SNP alone and cPTIO alone had no significant effect on MDA concentration and LOX activity in two cultivars.

### Effects of NO on antioxidant enzyme activities

**Figure 8:**
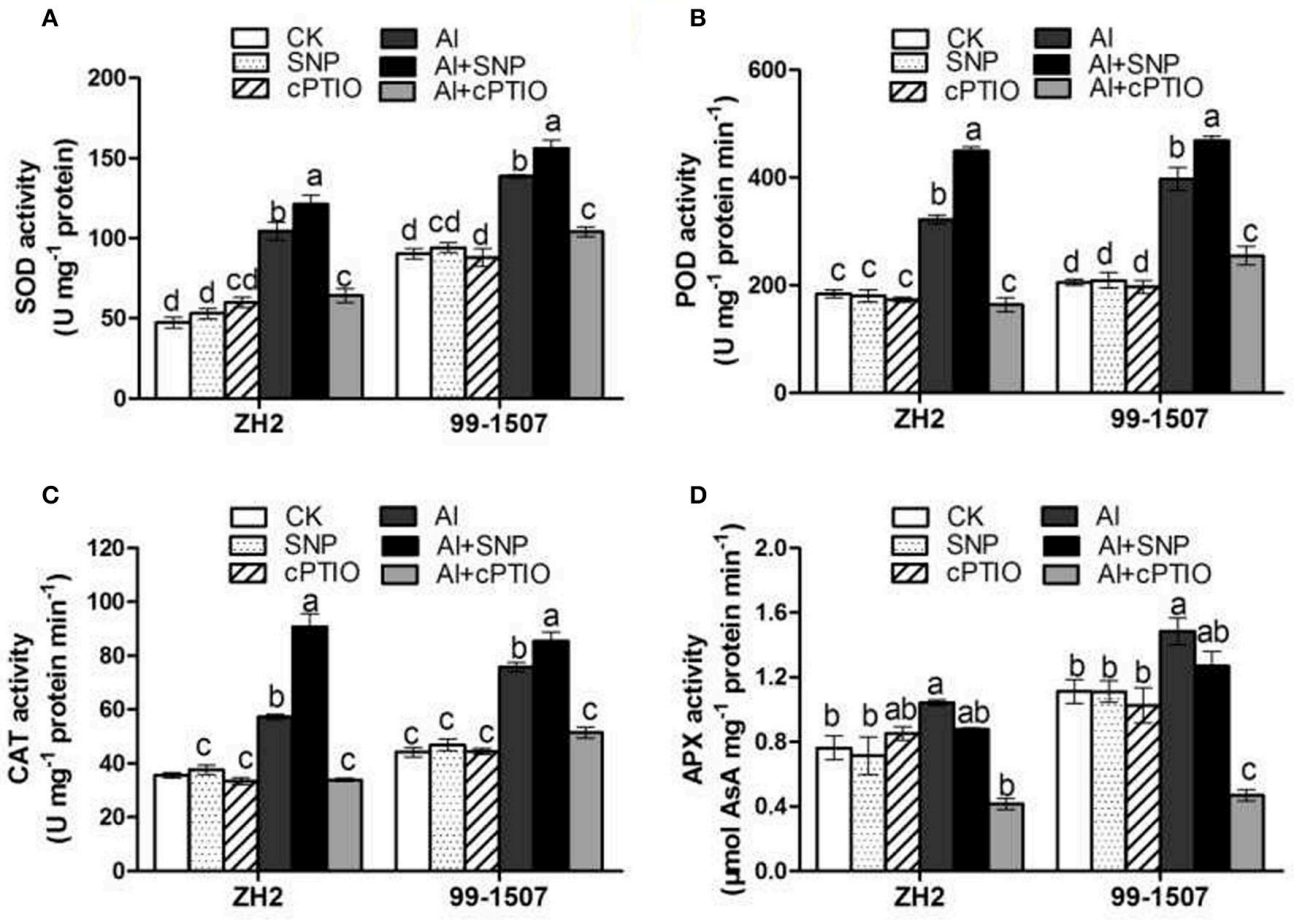
Effects of exogenous NO on antioxidant enzyme activities in root tips of ZH2 and 99–1507 with 100 μM Al treatment for 12 h. **(A)** SOD; **(B)** POD; **(C)** CAT; **(D)** APX. Values represent means ± *SD* (*n* = 3). Different letters indicate significant differences at *P* < 0.05.

Figure 8 showed that SNP and cPTIO alone had no significant effect on antioxidant enzyme activities in two cultivars. Compared with the control, Al stress increased the constitutive activity of all enzymes, while co-treatment Al with SNP further increased the activities of SOD, POD and CAT, but decreased the activity of APX. Contrary to the effects of SNP, co-treatment Al with cPTIO decreased significantly the activities of SOD, POD, CAT and APX in root tips of both cultivars. The Al-tolerant cultivar 99-1507 exhibited significantly higher levels of SOD, POD, CAT and APX than did the Al-sensitive cultivar ZH2 in both the control and Al-stressed roots (Figure 8).

### Effects of NO on PME activity

**Figure 9:**
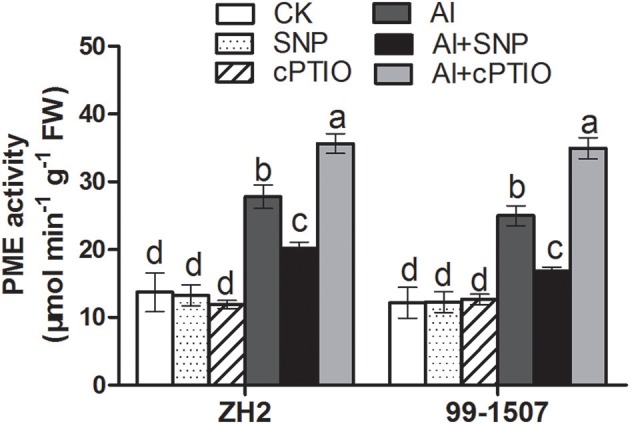
Effects of exogenous NO on PME activity in root tips of ZH2 and 99–1507 with 100 μM Al treatment for 12 h. Values represent means ± *SD* (*n* = 3). Different letters indicate significant differences at *P* < 0.05.

Al treatment resulted in a significant increase in PME activity in ZH2 and 99-1507 after 12 h of treatment (Figure 9). Compared with Al treatment alone, the PME activity of ZH2 and 99-1507 was reduced significantly by Al+SNP treatment, but was increased significantly under Al+cPTIO treatment (Figure 9). There was no clear difference between the cultivars in terms of PME response. Compared to the control, SNP alone and cPTIO alone had no significant effect on PME activity of two cultivars.

### Effects of NO on XET activity and *XTH-32* gene expression

**Figure 10:**
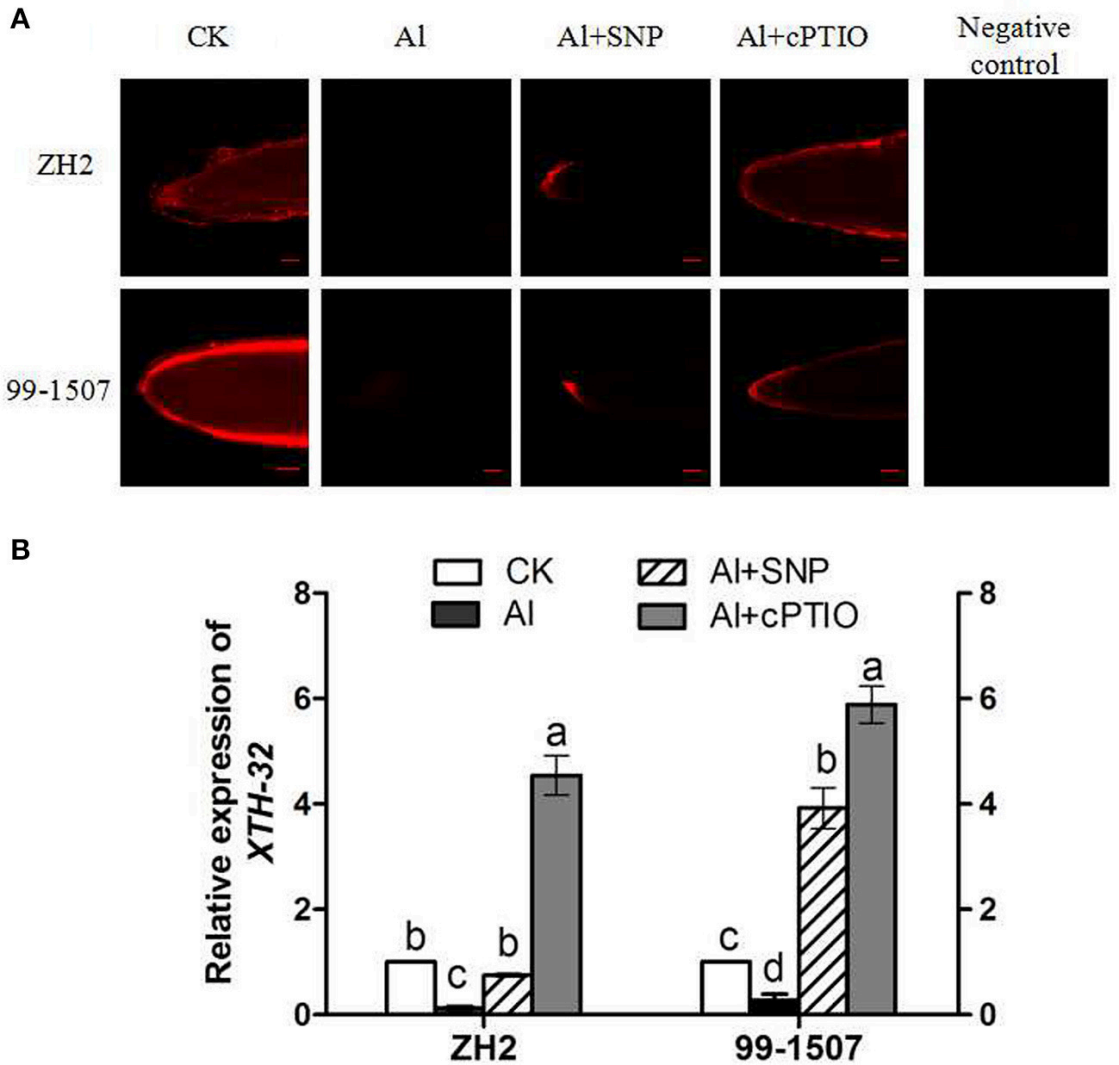
Effects of exogenous NO on XET activity **(A)** and relative expression (relative to that of *UBQ10R*) of *XTH-32* **(B)** in root tips of ZH2 and 99–1507 with 100 μM Al treatment for 12 h. Scale bars = 100 μm. Values represent means ± *SD* (*n* = 3). Different letters indicate significant differences at *P* < 0.05.

*In situ* localization of XET activity in peanut root tips was observed using the fluorescent substrate XGO-SRs. The negative control (incubation in MES buffer that lacked XGO-SRs) showed no autofluorescence in response to green light excitation (Figure 10A). Al stress significantly inhibited the XET activity of the two peanut cultivars. The addition of SNP slightly increased XET activity, while the addition of cPTIO significantly increased XET activity under Al stress.

Comparative transcriptomic analysis revealed that the expression of four genes encoding *XTH* (*XTH-1, XTH-2, XTH-23, XTH-32*) was significantly changed with prolonged Al exposure times, but the expression level of *XTH-32* showed the only statistically significant down-regulation in both cultivars, the other genes showed a transient increase in expression (data not shown). Transcriptional regulation of *XTH-32* plays an exclusive role in the expression of XET activity in response to Al stress. Compared with Al treatment alone, the addition of SNP increased the expression of *XTH-32* of ZH2 and 99-1507, but the increase was more remarkable in root tips of 99-1507. Exogenous cPTIO application also significantly increased the expression of *XTH-32* of ZH2 and 99-1507 (Figure 10B).

## Discussion

As a crucial signaling molecule in plants, NO plays a significant role in modulating physiological functions (Crawford and Guo, 2005). The effects of exogenous NO in alleviating Al toxicity and in decreasing Al accumulation in plants have been reported (Wang and Yang, 2005; Tian et al., 2007; Zhang et al., 2008). Exposure to Al resulted in the inhibition of root elongation in both the Al-tolerant cultivar 99-1507 and the Al-sensitive cultivar ZH2, with the inhibitory effect being much more pronounced in the latter (Huang et al., 2014a). In this study, we found that exogenous NO alleviated the Al-induced inhibition of root elongation and reduced Al accumulation in root apexes and CW (Figure 1). It was showed that the NO-promoted root elongation (in the Al+SNP treatment) was associated with a decrease in Al accumulation, both total Al and CW-bound Al (Figure 3). Compared with the control, the accumulation of Al in CW of peanut root tips was significantly increased, but the percentage of the total Al content in CW in 99-1507 was 16.2% higher than in ZH2 (Figure 3D). The results of Al adsorption and desorption kinetics in CWs demonstrated that the CW of ZH2 had a greater high adsorption ability to accumulate Al, SNP decreased Al adsorption in CWs and increased Al desorption in CWs in two cultivars (Figure 4). The results indicated that CW was the major site for Al accumulation in root tips, and the differences in Al adsorption and desorption in CW might cause the variation in Al tolerance, and NO decreasing Al adsorption in CW was one of mechanism of NO inhibiting Al-induced PCD. Similarly, it was found in rye and wheat that SNP treatment decreased significantly Al adsorption content of CW and alleviated the inhibition of Al on root elongation (He et al., 2007). Al binding to CW is an important pathway in the response of wheat root to Al toxicity, and reducing Al adsorption of CWs of root tip is important mechanism of exogenous NO alleviating Al toxicity (He et al., 2007).

Al stress causes molecular damage to plants, either directly, or indirectly through ROS formation, though the mechanism by which Al stress causes damage is not fully understood. However, increasing evidence has suggested that Al toxicity is due to oxidative damage, with Al toxicity altering the activities of antioxidative enzymes (Basu et al., 2001; Wang and Yang, 2005; Huang et al., 2014b). Our results showed that Al induced the accumulation of ROS, including ${\text{O}}_{2}^{\cdot -}$ and H_2_O_2_ in root tips of the two peanut cultivars, but that the accumulation of ROS in root tips of the Al-tolerant cultivar 99-1507 was less than that in ZH2 (Figure 5). Furthermore, Al enhanced the activities of antioxidant enzymes (including SOD, POD, CAT and APX) in root tips of the two cultivars, with the activities in the tolerant cultivar 99-1507 being higher than those in ZH2 (Figure 7), indicating that the Al-tolerant cultivar was better able to protect itself against ROS. Our results showed that exogenous NO alleviated Al toxicity by increasing antioxidant enzyme activities, thus reducing ROS concentrations and oxidative damage. It was suggested that lipid peroxidation is a relatively early symptom induced by the accumulation of Al (Yamamoto et al., 1997). Lipid peroxidation may be initiated either by ROS or LOX (Jalloul et al., 2002). We provided evidence that exogenous NO could significantly reduce LOX activity and MDA content, maintaining the stability of the cell membrane and thus alleviating Al-induced PCD.

In our previous study, we found that the exogenous NO donor SNP inhibited Al-induced PCD occurrence, while the NO specific scavenger cPTIO aggravated PCD occurrence, suggesting that exogenous NO negatively regulates the occurrence of PCD, while endogenous NO plays an important role in the development of PCD (He et al., 2017). Meanwhile, H_2_O_2_ might act as signal molecules in Al-induced PCD, with Al-induced ROS accumulation accelerating the production of PCD (Huang et al., 2014b). In this study, we provided evidence that endogenous NO triggered by Al might act as a stress molecule, quenching ROS especially signaling molecules H_2_O_2_ by activating antioxidant enzymes, regulating the occurrence of PCD. The removal of endogenous NO by cPTIO significantly decreased the activities of SOD, POD, CAT and APX, thus led to the increase of ROS.

NO has been shown to reduce Al toxicity by preventing oxidative stress in root tips (Wang and Yang, 2005; Giannakoula et al., 2010; Wang et al., 2010). The same protective effects of NO on heavy metal stress have also been found, such as exogenous NO alleviating Cd toxicity by enhancing the activities of antioxidant enzymes and reducing the accumulation of H_2_O_2_ in leaves of *Boehmeria nivea* (Wang et al., 2015). Similarly, it has been demonstrated that exogenous NO up-regulated the components of antioxidant defense machinery, namely CAT, POD, SOD and APX activity, in order to cope with Cu toxicity in root of *Panax ginseng* (Tewari et al., 2007). Recently, it was reported that exogenous NO increased the *S*-nitrosothiol (SNO) content of *Boehmeria nivea* leaves, simultaneously inducing an ameliorative effect against Cd toxicity by enhancing the activities of antioxidant enzymes (Wang et al., 2015). Yang et al. (2013) showed that exogenous NO mitigated Al-induced toxicity by modulating *S*-nitrosoglutathione reductase enzyme activity and efficiently suppressing the Al-induced increase in SNOs, implying that protein *S*-nitrosylation plays an important role in understanding the biological function of NO in plants tolerant to stress. Whether the underlying mechanism of NO in Al-induced PCD in peanut root tips is related to protein *S*-nitrosylation remains to be seen.

Not only does Al rapidly affect the properties of the plasma membrane, but its accumulation in the root also modifies the composition and properties of the CW (Horst et al., 2010). As one of the CW components, pectin, with its negative charge, has been proposed to be fundamental to the CW interaction with Al (Blamey et al., 1993). It has been demonstrated that Al stress increased the pectin content of CW in squash (Van et al., 1994), maize (Eticha et al., 2005), and wheat (Sun et al., 2016). Depletion of exogenous NO acts to decrease Al accumulation in CW, mainly by modulating the enhanced methylation of pectin (Sun et al., 2016). The lower level of pectin methylation in CW, the greater Al accumulation was in CW and root tips (Darley et al., 2001). PME could reduce the degree of pectin methylation (Zhang et al., 2011). In the present study, we found that Al induced an increase in PME activity in both cultivars (Figure 8). This was consistent with the higher Al accumulation measured in ZH2, demonstrating that the increased PME activity induced by Al was involved in the regulation of Al adsorption by CW, thereby accelerating Al toxicity. The addition of exogenous NO significantly reduced PME activity, leading to higher pectin methylation and less binding sites for Al^3+^, preventing Al from entering root tip cells.

XET has been proposed to play a predominant role in cell expansion by cleaving xyloglucan molecules endolytically and being responsible for the incorporation of newly-synthesized xyloglucans into the wall matrix (Darley et al., 2001). Yang et al. (2011a) found that Al greatly inhibited XET activity within 30 min of exposure, indicating that the reduction in XET activity could play an important role in Al-induced root growth inhibition. Under Al stress, XET activity and the expression of *XTH-32* in root tips of peanut were significantly reduced (Figure 10). However, only ZH2 could maintain XET activity at a certain level within 4 h of Al stress when PCD was clearly induced by Al treatment at 100 μM for 4 h, but only weakly in 99-1507 (Huang et al., 2014a). It demonstrated that XET activity might be involved in Al-induced root growth inhibition of peanut root tips and thus participate in the regulation of Al-induced PCD. The addition of exogenous NO significantly enhanced XET activity and the expression of *XTH-32* in peanut root tips, achieved extensibility of the CW, and thus alleviated Al-induced PCD. Whereas the addition of cPTIO under Al stress also significantly enhanced XET activity and the expression of *XTH-32* in peanut root tips, suggesting the different roles of endogenous NO in Al-induced PCD.

Over expressing *XTH* genes, likely to be related to a decreased uptake and storage capacity of the root. A mutant of *XTH15*, which make a contribution to cut or cut and rejoin XyG chains, demonstrates lower endogenous auxin levels and CW Al, but higher symplastic Al than the WT *Arabidopsis* (Zhu et al., 2013). Over expressing *AtXTH21*, showed an improved frost tolerance in transgenic *Arabidopsis* plants (Shi et al., 2014). Similarly, the transgenic plants exhibited an increased tolerance to severe water deficit, confirming that *XTH* is involved in drought tolerance (Cho et al., 2006; Choi et al., 2011). The expression behavior of *OsXET9* was differently affected by drought in rice depending on tissue and development stage (Dong et al., 2011). Qu et al. (2006) found that endogenous NO induced by ultraviolet-B inhibited the xyloglucan-degrading activities in cell walls which led to the inhibition of pea stems elongation. Liu et al. (2016) found that cell wall invertase (CWIN) involved in the suppression of LMHS (long-term moderate heat stress)-induced PCD in transgenic tomato in a ROS-independent manner. It is possible that NO might have responded to CWIN-mediated Suc catabolism and Glc signaling to alleviate PCD under heat stress. In addition to being a signaling molecule, exogenous NO might involve in the regulation of extensibility of CWs by modifying the chemical properties of the CW, changing the activity of key enzymes and the expression of key genes by altering intracellular NO concentration, and thus alleviating Al-induced PCD in peanut root tips.

## Conclusion

All of these results showed that NO decreased Al adsorption in CW, decreased PME activity and ROS contents, increased the activities of SOD, CAT, POD and the expression of *XTH32*. It reveal the importance of exogenous NO in protection against the deleterious effects of Al stress and in the amelioration of Al-induced PCD in root tips of peanut, and may possibly suggest the mechanism by which NO helps plants withstand Al stress. Firstly, we found that exogenous NO might increase the antioxidant content and antioxidative enzyme activity to enhance antioxidant defense against Al stress. Secondly, exogenous NO might decrease the content of pectin and increase the XET activity of CW, which determined the Al-binding capacity of CW, leading to lower Al accumulation in CW, maintaining the extensibility of the CW. In the meanwhile, the results can be used to breed new cultivars with high Al tolerance by molecular breeding. In fact, NO may be used for ameliorating Al toxicity on crop in practice as well as slaked lime.

**Figure 11:**
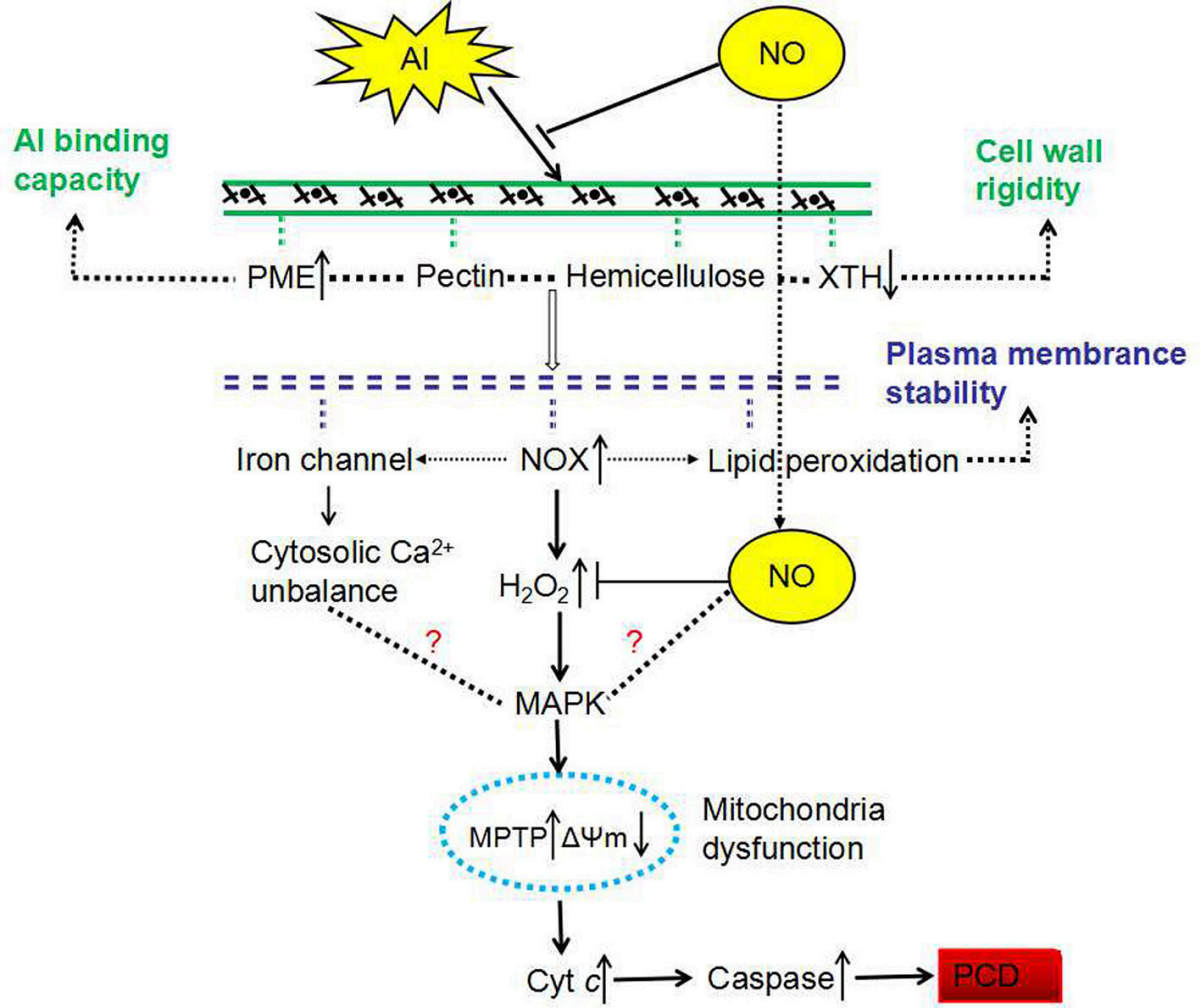
Proposed mechanism of NO response to Al-induced PCD in peanut root tips. The up and down arrows represent increases and decreases, respectively. NOX, NADPH oxidase; MAPK, mitogen-activated protein kinase; MPTP, mitochondrial permeability transition pore; ΔΨm, inner membrane potential; Cyt *c*, cytochrome *c*.

Taken together, based on our previous and the present findings (Zhan et al., 2013, 2014; Huang et al., 2014a; Yao et al., 2016; He et al., 2017), an improved model was proposed to reveal the regulation mechanism of NO in Al-induced PCD (Figure 11). The involvement of NO on preventing Al-induced PCD mechanisms mainly includes: (1) Regulating the activity of PME and XET, and the gene expression level of *XTH-32*, reducing Al binding sites, maintaining the relaxation and extensibility of CW; (2) Maintaining the integrity of cell membrane by reducing ROS production and alleviating lipid peroxidation; (3) Regulating the ion channels of cell membrane and maintaining the balance of cytosolic calcium ion; (4) Maintaining mitochondrial function, reducing the release of Cyt *c*, reducing the activity of caspase-3-like, and thus preventing the occurrence of PCD.

## Author contributions

L-FH: conceived the research and designed the experiments; JZ: designed the experiments and revised the manuscript; C-LP: conducted the DAPI and hematoxylin staining, determined NO content, antioxidant enzyme activity, ROS, wrote the manuscripts and analyzed the data; S-CY: conducted the detection of MDA content, PME activity, XTH activity and gene expression by qRT-PCR; W-JX: conducted the detection of root growth, cell death and Al accumulation; S-ZL: conducted the Al adsorption and desorption kinetics assay; Y-LW: conducted the detection of Al content in CW; A-QW and DX: analyzed the data. All authors read and approved the final manuscript.

### Conflict of interest statement

The authors declare that the research was conducted in the absence of any commercial or financial relationships that could be construed as a potential conflict of interest.
